# Multi-Techniques Analysis of Archaeological Pottery—Potential Pitfalls in Interpreting the Results

**DOI:** 10.3390/molecules30244732

**Published:** 2025-12-10

**Authors:** Lidia Kozak, Andrzej Michałowski, Yana Tkachenko, Jędrzej Proch, Jarosław Jasiewicz, Przemysław Niedzielski

**Affiliations:** 1 Faculty of Chemistry, Adam Mickiewicz University in Poznań, 8 Uniwersytetu Poznanskiego Street, 61-614 Poznań, Poland; 2 Faculty of Archaeology, Adam Mickiewicz University in Poznań, 7 Uniwersytetu Poznanskiego Street, 61-614 Poznań, Poland; 3 Interdisciplinary Research Group Archaeometry, Faculty of Archaeology and Faculty of Chemistry, Adam Mickiewicz University in Poznań, 7-8 Uniwersytetu Poznanskiego Street, 61-614 Poznań, Poland; 4 Faculty of Geography and Geology, Adam Mickiewicz University, 10 Bogumiła Krygowskiego Street, 61-680 Poznań, Poland

**Keywords:** archaeometry, pottery, pre-Roman Iron Age, XRF, FTIR, UV-Vis, HPLC-ICP hrOES, results interpretation

## Abstract

This article presents the results of an analysis of ceramics from archeological sites. The main goal of the study was to determine the elemental composition of ceramics using XRF. This study was conducted in two stages. The first stage involved the analysis of complete vessels from the museum exhibition. The second involved the interpretation of the results obtained from the first stage. In the second stage, 30 samples obtained by dividing a single fragment of a ceramic vessel were analyzed. The results (results scattered due to material heterogeneity) were compared with the results of analyses of a large group of ceramic samples from a similar period. To supplement the information about the ceramic material studied, destructive analyses were also performed (after grinding the aforementioned 30 samples), namely mineral composition using FTIR (to determine the raw materials) and iron speciation using UV-Vis and HPLC-ICP hrOES (to determine the firing method). The results obtained indicated that limiting the research to the most-used non-destructive procedures in archaeometry can lead to misinterpretation. Although the presented study concerned archaeological objects, it can be considered in the context of research on other materials.

## 1. Introduction

Chemical composition studies are among the basic tools used in archaeometric research. Determining elemental content can lead to the identification of the materials from which the artifacts were made. Non-destructive techniques such as XRF are used in the study of the elemental composition of archeological artifacts [1,2] due to their often unique nature. Examples of research include analysis of various objects, e.g., bricks [3], coins [4], jewelry [5], mortars [6,7], pigments [8], and paintings [9]. The XRF technique is also used in the analysis of pottery from archaeological sites [10,11]. Analyses of the elemental composition of ceramics provide information allowing the interpretation of the origins of pottery [12] and the technology of their production [13]. Due to the availability of portable spectrometers, such measurements can also be performed directly at an archaeological site [14]. As mentioned above, choosing a non-destructive method seems to be the best solution in the study of archaeological artifacts due to the historical value of the objects. However, in the study of pottery, which is present in mass in archaeological sites, procedures that require the destruction of a fragment of the studied object are also used. In studies using ICP-OES and ICP-MS spectrometry, it is possible to determine the elemental composition of archaeological ceramics [15,16,17] in a range that significantly exceeds the possibilities of non-destructive techniques. The use of various procedures for transferring the components of the sample to the solution allowed the determination of not only the total content of elements in the ceramics after melting and acid digestion [18,19] or hydrofluoric acid decomposition [20] but also the possibility of the migration of the ceramics components during their deposition in the archaeological site using weak acid extraction [21].

Another important issue in archaeometric research is attempting to identify the technological processes by which the artifact was produced. In ceramics research, the identification of iron forms will play a particularly important role. The study of the content of iron forms (i.e., the study of iron speciation [22]) potentially indicates the manufacturing processes of pottery [23]. The ratio of the content of the iron forms (Fe(III)/Fe(II)) varies depending on the method of firing the ceramics [24]. The coefficient Fe(III)/Fe(II) assumes high values (over 100) for ceramics fired under an oxidizing atmosphere and low values (below 10) for ceramics fired under a reducing atmosphere. Although the method of firing ceramics is determined based on the color of pottery shards, studies of iron speciation support this process and make the estimation of the firing method more objective [24,25]. In the study of iron speciation, many procedures and analytical techniques can be used, from colorimetric procedures based on UV-Vis measurements [26] to the use of hyphenated chromatographic techniques [25]. Due to the high degree of Fe^3+^ substitution in the structure of the clay mineral and therefore also in the ceramic material, the HPLC-ICP OES technique becomes a useful tool for iron speciation studies [27]. The HPLC-ICP OES technique can also be used as a reference technique for much simpler colorimetric measurements [25].

Identifying the minerals present in the tested material allows for its better identification. Among the procedures used to determine the presence of minerals in ceramics, the FTIR technique is gaining prominence. FTIR is a technique used to identify the material from which archeological artifacts were made. The technique allows the direct determination of the type of bonds occurring in chemical compounds and the indirect determination of (using spectral libraries), among others, the type of chemical compounds that make up the material (minerals, rocks, pigments, organic materials, etc.). The FTIR technique is used in the study of archaeological artifacts such as gemstones [28], wall paintings [29], and fabrics [30]. The FTIR technique has been successfully used in the geochemical study of clay, hence its application in the study of archaeological ceramics. The FTIR technique was used to identify the minerals present in the original material from which pottery was made [31,32] or to identify technological processes [33,34]. Some of the studies on ceramics using the FTIR technique concern the analysis not of the pottery itself but, for example, of the presence of residues of the substances stored in them [35]. Importantly, the FTIR technique is treated both as an individual research tool [36] and more often as an additional technique, supplementing the information obtained in other multi-technique analyses [37,38]. It should be emphasized that in most of the presented studies, several analytical techniques were used, which allowed for better characterization of the analyzed material. The obtained information, complementing each one another, allowed for a much better interpretation of the results.

This article presents archaeometric studies of archeological ceramics conducted in three stages. In the first stage, the concentration of selected elements was mapped (using XRF) on the surfaces of complete vessels from the museum exhibition. In the second stage, an additional experiment was conducted to interpret the results of this mapping. A fragment of another vessel, divided into 30 pieces, was analyzed. The content of the selected elements in these samples was also determined using a non-destructive method (XRF). The third stage of the study was complementary. After appropriate sample preparation (grinding), the mineral composition (FTIR) and iron speciation (UV-Vis, HPLC-ICP hrOES) were determined. This allowed for a better characterization of the examined ceramic fragment (divided into 30 pieces).

## 2. Results and Discussion

### 2.1. Non-Destructive XRF Analysis of Whole Vessels

For the first vessel, the analysis was carried out at 237 points, and for the second vessel at 155. The difference in the number of measurements was due to the different shape of the vessels and the fact that vessel 2 was not complete (Figures S1 and S2). The mass fraction of selected elements (Si, Al, K, Mg, Fe, Ca, Ti, Ba, Mn) was determined by XRF at each of the points. The results obtained were presented graphically in the form of maps (Figures S1 and S2 in Supplementary Materials) for each of the vessels. Concentration maps showed that the content of each element varies for different parts of the vessel. These changes did not depend on the part of the vessel tested and were different for both vessels. Changes in the concentrations of elements are presented numerically (minimum and maximum values, mean, median, standard deviation, and relative standard deviation, and max/min ratio) in Table 1. Parameters describing the central value (mean and median) for all elements and for both vessels have similar values. This demonstrates the nature of the data distribution close to the normal distribution. In turn, the dispersion of the results, determined by the relative standard deviation, differed for individual elements, amounting to 10–34% for the first vessel and 14–68% for the second. The RSD values were greater for the results obtained for the second vessel (the exception was the RSD for the results of Fe and Mn contents). The second parameter describing the dispersion of the results was the max/min ratio. This parameter determined the size of the range within which the results occurred. For the first vessel, the values of the max/min ratio ranged from 1.6 (for K) to 23 (for Ba), and for the second vessel from 2.5 (for Mn) to 43 (for Ba). Such high values of the max/min ratio mean that the concentrations of the elements measured at various points in the vessel may differ several dozen or even over a hundred times.

**Figure 1:**
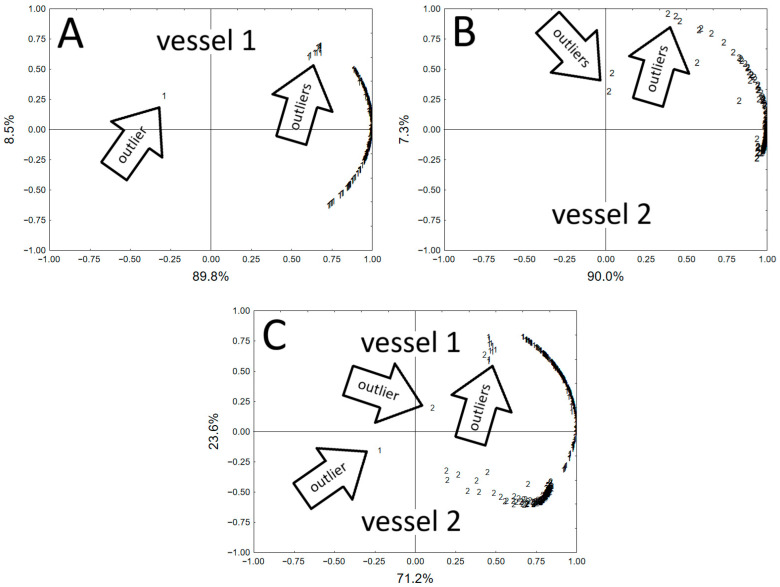
Principal components analysis visualization for XRF results. (**A**)—Vessel 1 (two components explain 98.3% of the variability). (**B**)—Vessel 2 (two components explain 97.3% of the variability) (**C**)—Both vessels 1 and 2 (two components explain 94.8% of the variability).

The results obtained were also subjected to exploration analysis (PCA). The analysis was performed for the results of the contents of Si, Al, K, Fe, Ca, Ti, Ba, and Mn for each vessel separately and for the set of results obtained for both vessels (Figure 1). The exploration analysis showed the differences in the chemical composition of both vessels. Additionally, the exploratory analysis allowed for the identification of points on the surfaces of both vessels where the concentrations of the analyzed elements differed significantly from the rest [2]. These observations are similar to the results of statistical analyses in archaeometric studies of many pottery fragments [3,18]. Based on the graphical presentation of the PCA, it would be possible to identify outliers that could identify samples from vessels of other proveniences (imports) or produced using different pottery technologies [1]. The fact that whole vessels were analyzed in these studies indicates the danger of directly interpreting the results of non-destructive XRF analysis. The non-homogeneity of ceramic materials causes a significant diversification of the results of the analysis.

However, without considering the outliers from the main groups identified in Figure 1 as vessel 1 and vessel 2, PCA allows for inferences in the field of archaeology. The first vessel and the single shard from the pot are coarse-made vessels that are technologically similar. The second vessel, on the other hand, is a delicately made vessel with better-cleansed clay and differs from them technologically. Also, despite the similarity of macroscopic forms of both vessels, vessel 1 is chemically more similar to the pot, because both are in the same technological group. Therefore, the technology of making the vessel is determined, and the similarity in the production of coarse-ware pottery is confirmed. Therefore, fine-ware pottery was produced using a different technology and the following were noted:

- Different clays were used (or a combination of coarse clays—that is, a matrix from various outcrops).
- The type of admixture was important (medium-fine-grained here).
- The firing temperature may be important—vessel 2 had a different color and turned gray, so either a reduction or a pseudo-reduction firing, and there was certainly oxygen available.
- A combination of the above factors.

### 2.2. Non-Destructive XRF Analysis of Pottery Fragment

A fragment of the damaged vessel was divided into 30 parts, and each of them was analyzed for both the outer and inner parts (Figure S3, Supplementary Data). The analysis was conducted on both sides of the pottery fragments, representing the outer and inner parts of the vessel. They served different functions (decorative and utilitarian), and their standards of workmanship and contact with external factors (fire) were different, as evidenced by the analysis results. Information on the concentration of 30 elements was obtained (a total of 900 results for each side of the sample). The simple descriptive statistic of the concentration of the selected 8 elements is presented in Table 2 (minimum and maximum values, mean, median, standard deviation, relative standard deviation, and max/min ratio).

The mean and median values, as parameters describing the central value of the elements, were similar for most of the results obtained for the analysis of pottery fragments. This demonstrates that data distribution was close to the normal distribution. The relative standard deviation was different for individual elements, amounting to 13–60% for the outer part of the pottery fragment and 11–67% for the inner part of the pottery fragment. The next parameter describing the dispersion of the results was the max/min ratio. This is a simple parameter defining how many times the minimum value is smaller than the maximum value of the concentration of a given element. For the outer part of the pottery fragment, the values of the max/min ratio ranged from 2.0 (for Si and Fe) to 39 (for Ba), and for the inner part of the pottery fragment, from 1.5 (for K) to 28 (for Ba). High values of the min/max ratio indicated a large variation in the concentrations of the determined elements, reflecting the heterogeneity of the tested object.

**Figure 2:**
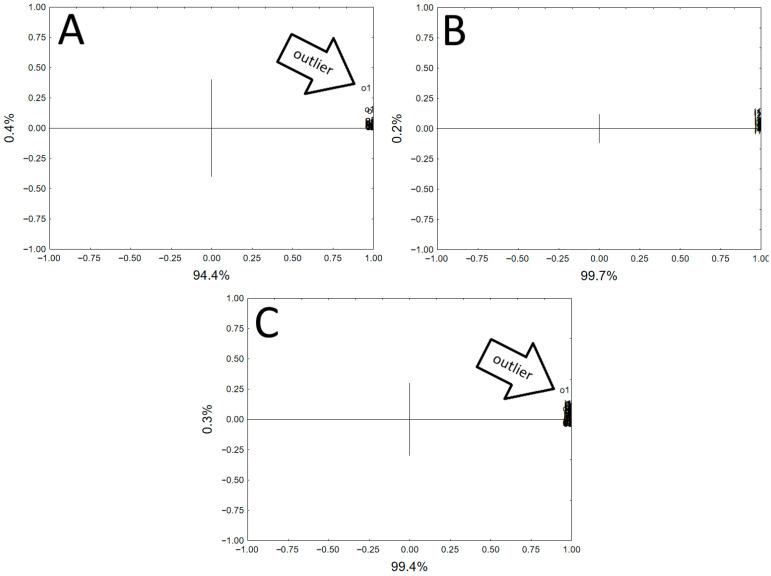
Principal components analysis visualization for XRF results. (**A**)—Outer part of pottery fragments (two components explain 99.77% of the variability). (**B**)—Inner part of pottery fragments (two components explain 99.91% of the variability). (**C**)—All pottery fragments (two components explain 99.70% of the variability).

Similar to the tests of whole vessels, the results of the analysis of the pottery shards were subjected to statistical analysis (PCA). Statistical analysis was performed for the results of the concentrations of the elements to be determined, separately for the outer and inner parts of the pottery fragment, and for all results (Figure 2). The statistical analysis indicated the homogeneity of the samples, although several samples with a different chemical composition were identified for the outer surface of the pottery shard. As with the analysis of whole vessels, it is possible to identify samples that differ in chemical composition, which could be interpreted as samples from vessels made of different materials, e.g., imports. However, the nature of these studies (the samples come from one fragment of the vessel) precludes such an interpretation of the results. As for the analysis of whole vessels, the results obtained indicate the risk of misinterpreting the results of the analysis. The non-homogeneity of the ceramic material, visible in microscopic photographs (Figure S4, Supplementary Data), is the reason for the large dispersion of the obtained results of the analysis.

Interestingly, the max/min ratio values obtained in the study of whole vessels and a larger fragments of a vessel divided into 30 parts do not differ substantially from the results obtained from a large collection (*n* = 100) of archeological pottery fragments (also pre-Roman Iron Age, for six elements (Ba was not determined, Mn concentrations were not taken into account for comparison) Table 3 based on [2]). This fact highlights the problem of interpreting analytical results. Assessing the variation in the chemical composition of a set of pottery fragments must consider the material’s inhomogeneity, determined for well-identified individual objects. Otherwise, there is a risk of misidentifying workshop differences or potential ceramic imports.

Studies of the chemical composition of ceramics and exploratory analysis (PCA) of the results indicated that the presence of statistically significant differences in sample composition is not sufficient to interpret the cause of these differences. Identifying samples with different compositions as “outliers” can lead to their misidentification as evidence of imports or technological differences. Any conclusions regarding differences in the chemical composition of samples must be preceded by an analysis of their “natural” heterogeneity. Only the existence of differences beyond the “natural” properties of the material allows for a deeper interpretation of the differences and inferences regarding the distinct origins or formation of artifacts.

### 2.3. Destructive Analysis of Pottery Fragments

Non-destructive procedures are the best in the study of archaeological artifacts. However, in the study of ceramics, a material that occurs in large amounts in archaeological sites, destructive tests are also acceptable, causing a slight (loss of about 1–2 g) damage to the pottery fragment. Such research has been provided in archaeometry for many years [21,39,40]. The obtained powdered samples were subjected to FTIR analysis and then to HCl extraction procedures described above.

#### 2.3.1. Iron Forms in Pottery

**Figure 3:**
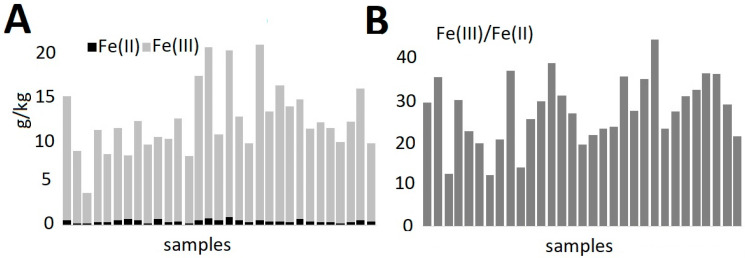
Iron speciation in pottery fragments: (**A**)—concentration of Fe(II) and Fe(III), (**B**)—Fe(III)/Fe(II) ratio.

The occurrence of the iron forms Fe(II) and Fe(III), or the ratio of the content of these forms (Fe(III)/Fe(II)), carries information about the technology of producing ceramic vessels [23,24]. In previous studies [25], it was indicated that low values of the Fe(III)/Fe(II) ratio probably show reduced firing conditions, while high values indicate oxidizing conditions. Moreover, in previous studies, a very large variation in the coefficient (1–485) for a set of 126 pottery fragments from one archaeological site was found. The reasons for this variation in the content of iron forms were indicated as high variability and lack of repeatability of the conditions of the technological process. In this study, the form Fe(III)—mean 11,900 mg/kg—was found to be advantageous over the form Fe(II)—mean 460 mg/kg—with RSDs of 32% and 34%, respectively. The values of the Fe(III)/Fe(II) ratio ranged from 12 to 44—mean 27, RSD 28%. The relatively small differentiation (Figure 3) of the coefficient indicates similar firing conditions for all samples. Referring to the literature [23,26], the Fe(III)/Fe(II) ratio indirectly describes the conditions under which the ceramics were fired. Its value can range from a few to over two hundred [25]. Low values of the Fe(III)/Fe(II) ratio—around 20—indicate the predominance of reducing conditions for firing the ceramics, and high values (100 and more) the predominance of oxidizing conditions [24]. The average value of the Fe(III)/Fe(II) ratio of 27 ± 7 obtained in this study allows for the indication of both the potential firing under reducing conditions and the homogeneity of the method of the tested ceramic fragments (which is not surprising, as they constitute parts of a larger ceramic fragment).

#### 2.3.2. Mineral Composition Analysis Using ATR-FTIR

**Figure 4:**
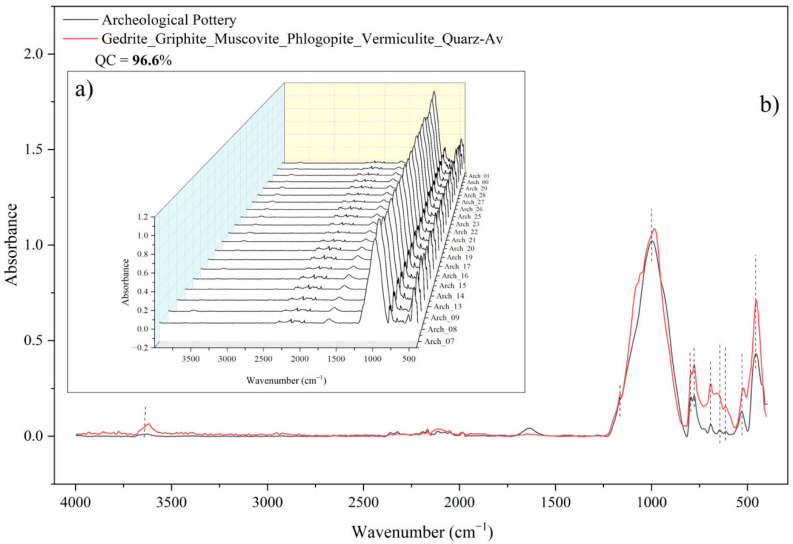
FTIR absorbance spectra of samples from the pottery fragments: (**a**) comparing the spectra of the pottery fragments; (**b**) comparing the averaged spectrum of pottery fragments (black spectrum) to the averaged spectrum of the standard spectra of minerals (red spectrum).

The samples were subjected to three analyses, each according to the above-described procedure, with the following averaging of spectra. A total of 60 absorbance spectra were obtained. The spectra for all tested samples were identical (Figure 4a), which confirms the identity of the mineral composition from which the fragments were made. This is particularly evident in the context of their origin from a single vessel. The analysis of the infrared spectra revealed no differences and showed the homogeneity of the original material from which the vessel was made. Comparison of the averaged sample of pottery to the averaged standard spectra from the infrared library demonstrated their similarity with 96.6% accuracy (Figure 4b). Hence, the mineralogical composition of the investigated samples, according to the infrared library, was as follows: gedrite, griphite, muscovite, phlogopite, vermiculite, and quartz.

The ATR-FTIR spectra of pottery showed very weak peaks at 3668, 3648, and 3629 cm^−1^. These peaks represent the stretching vibrations of O-H bonds in clay minerals, probably from kaolinite. According to [41], kaolinite is characterized by the presence of four peaks in the high frequency region, namely at 3695, 3669, 3653, and 3620 cm^−1^, with the Al_2_OH bending band near 914 cm^−1^. One possible explanation is that the ceramics were subjected to heat treatment during the vessel’s manufacture, and these peaks could shift. This is confirmed with the results obtained by [42], which highlighted a decrease in intensity and a shift in these peaks with rising temperature. The peak at 1638 cm^−1^ was assigned to H-O-H bending deformation of adsorbed water.

**Figure 5:**
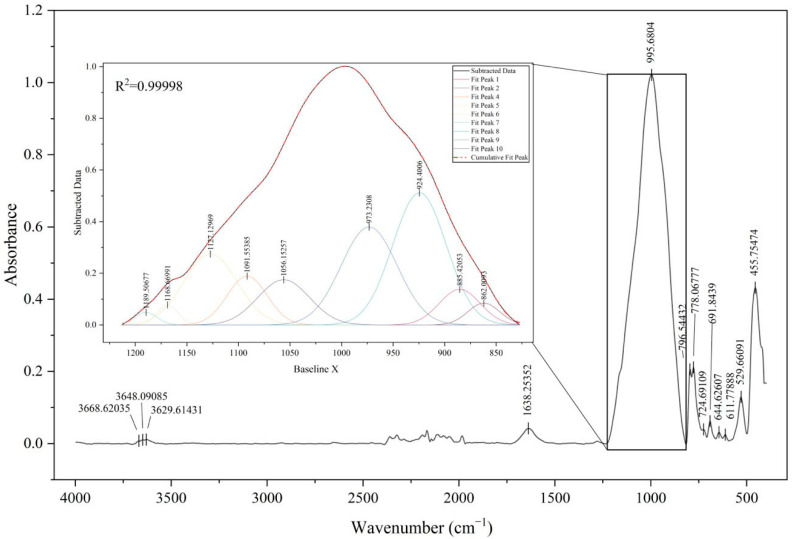
The peak fitting result of the archaeological pottery’s ATR-FTIR spectra in a selected range using Gaussian peaks with a subtracted baseline.

As shown in Figure 5, the peak with a maximum of 995 cm^−1^ (Si-O stretching) represents an overlapping signal from different minerals. Therefore, the peak fitting procedure was performed (Figure 5). According to this, we obtained the following peaks: 862, 885, 924, 973, 1056, 1091, 1127, 1168, and 1189 cm^−1^. The cumulative fit peak was obtained with an R^2^ value of 0.99998. The absorbance band at 924 cm^−1^, assigned to the Si-O-Si stretching vibration, is characteristic of muscovite [43]. The band at 974 cm^−1^ corresponds to the Si-O/Si-O-Al stretching mode attributed to phlogopite [44]. Peaks observed at 1056 cm^−1^ and 1168 cm^−1^ represent Si-O-Si vibrations associated with microcline and quartz, respectively [45,46]. The 1091 cm^−1^ band is indicative of chlorite or possibly griphite/gedrite [47]. The absorbance at 862 cm^−1^ is assigned to Al-O-H/Fe-OH-Al bending vibrations in kaolinite [45,48]. An additional band at 1127 cm^−1^ was observed and probably corresponds to kaolinite after interaction with organic matter, which contains N-N bonds [48]. The obtained peak at 885 cm^−1^, with a weak shoulder at 724 cm^−1^ and a weak signal near 1415 cm^−1^, indicates recarbonated calcite or another carbonate mineral [49].

The frequency range between 800 and 400 cm^−1^ is dominated by vibrational modes associated with Si-O and metal-oxygen (Me-O) bonds. In the FTIR spectrum of archeological pottery, the absorption bands found at 796, 778, 691, 644, 455 cm^−1^ are characteristic of Si-O-Si(Al) and O-Si-O stretching vibrations. The band at 795 and 776 cm^−1^ can be attributed to symmetric Si-O vibrations in quartz, the presence of which is further confirmed by an out-of-plane bending deformation at around 693 cm^−1^ and a peak at around 450 cm^−1^ [50]. The absorption band observed at 644 cm^−1^ corresponds to microcline [51], while the lattice vibration mode at 611 cm^−1^ indicates goethite [52]. In addition, the peak recorded at 529 cm^−1^ can be assigned either to Si-O-Al bending vibration or to contributions from an iron-bearing phase [50,53].

Summarizing all of the above, the possible mineralogical composition of the investigated vessel consists of the following mineral phases: gedrite, griphite, muscovite, phlogopite, vermiculite, quartz, kaolinite, chlorites, carbonate minerals, microcline, and goethite. These findings are consistent with the previous studies [32,46,54,55].

#### 2.3.3. Characteristics of Ceramic Material

The study of the content of the iron forms and mineral composition showed, in accordance with the methodology, that all analyzed pottery samples came from one vessel. The results of the concentration of elements in ceramics are different for each analytical procedure. In each of the procedures, a different feature of the analyzed material was tested. XRF analyzes the total content of elements on the surface of the ceramics. Depending on the density of the sample and the determined element (characteristic energy), the surface layer of pottery at different depths was analyzed. In addition, the size of the sample surface for which the measurement was made was limited (in these tests, the diameter of the test surface was about 8 mm). Hence, local material heterogeneities influenced the measurement result. In the case of destructive analyses, preceded by grinding the sample, the method of preparation for analysis determined the feature of the object to be tested. In turn, using HCl extraction, the content of iron forms in the acid-leachable fraction was determined. This fraction was not related to the aluminosilicate core and may be exchanged with the external environment [56], e.g., when ceramics remain in an archaeological site. Thus, the results do not directly describe the composition of the primary material and may be different for fragments of the same vessel, as observed in this study.

## 3. Experimental

### 3.1. Analyzed Pottery Description

Three ceramic artifacts were selected for analysis (Figures S1–S3). Two of them were well-preserved pots. The third object was a large fragment of pottery from another vessel. The archaeological material intended for archaeometric research was obtained from a well-examined, developed, and published settlement dated to the turn of the older and younger pre-Roman Iron Age (turn of the 3rd/2nd century BC) from the site Grabkowo 7, district Włocławek, voivodeship Kuyavian-Pomeranian [57]. Two almost completely preserved vessels of the same macromorphological form but with different technological features and one single ceramic fragment, technologically similar to the first vessel, were selected for the study. Both examined vessels represent vase-shaped forms. At site 7 in Grabków, 16 specimens were classified into this category of vessels. They were characterized by an out-curved, flared rim and a high bend of the body. In both specimens taken for analysis, the bases were marked out in a low leg. Both vessels were equipped with band-like handles, although most of the other vessels from Grabków were vessels without handles. Vessel 1 was from feature 125, which is the remnant of a sediment cavity. The vessel was intact, with a partially damaged rim tilted outward in a flanged shape and a damaged band-shaped handle. Its orifice diameter was 12 cm, the base diameter was 5.5 cm, and the height was 8.4 cm. The outer surface of the vessel was naturally smooth, multicolored (light brown, brown-red, gray-brown, beige), and medium-grained. Vessel 2 was obtained from feature 59, which was a charcoal pile. The vessel was almost completely reconstructed and had an out-curved, rounded rim; a rounded flat base; and a slightly marked, fully preserved tape-shaped handle. Its orifice diameter was 15.5 cm, the base diameter was 5.5 cm, and the height was 10.2 cm. The outer surface was carefully smoothed, matte, dark gray-dark brown, and fine-grained. A single piece of pottery came from a large vessel, most likely from a pot. The outer surface was smooth, light brown in color, with a medium-grained admixture. Within the excavated area, the features from which the vessels were located were about 110 m from each other. Feature 125 was in the northeastern zone of the site, and feature 59 was in its southern part. Between these two features an analyzed single piece of pottery was discovered.

### 3.2. Reagents

Ultra-pure argon (N–5.0, purity 99.999%), obtained from Linde (Kraków, Poland), was used as the plasma gas for HPLC–ICP hrOES. The ultra-pure deionized water (≥18 MΩ cm resistivity) obtained from a Milli-Q water purification system (suprapure grade, Millipore, Burlington, MA, USA) was used for all solutions. For sample extraction and digestion, 65% nitric acid, 40% hydrofluoric acid, 30% hydrochloric acid, and boric acid (Merck, Darmstadt, Germany) were used. For UV-Vis and HPLC-ICP hrOES analysis, stock standard solutions were prepared by dissolving in water appropriate amounts of ferrous ammonium sulfate hexahydrate and ferric ammonium sulfate dodecahydrate obtained from Acros Organics (Waltham, MA, USA). For colorimetric UV-Vis analysis, 2,2′-bipyrydyl, thiocyanate, acetate buffer, and hydrochloric acid (analytical grade, POCh, Gliwice, Poland) solutions were used. For HPLC-ICP hrOES analysis, a PDCA eluent was prepared by mixing appropriate volumes of pyridine–2,6–dicarboxylic acid (PDCA) and formic acid (HCOOH) (Sigma-Aldrich, St. Louis, MO, USA).

### 3.3. Instruments

The X-ray Tracer III XRF (Bruker AXS, Madison, WI, USA) spectrometer, characterized by the following technical parameters—voltage range 4 kV–45 kV, current 1 µA–45 µA, and Silicon Drift Detectors (SDD)—was used. The spectrometer was equipped with a vacuum pump. The estimated detection limits were 120 mg kg^−1^ for Si, 300 mg kg^−1^ for Al, 30 mg kg^−1^ for K, 800 mg kg^−1^ for Mg, 10 mg kg^−1^ for Fe, 23 mg kg^−1^ for Ca, 15 mg kg^−1^ for Ti, 30 mg kg^−1^ for Ba, and 15 mg kg^−1^ for Mn. Two built-in calibrations were used: Bruker Mudrock Major (instrument parameters: 15 keV, 25, μA, vacuum < 17 Torr) and Bruker Mudrock Trace (parameters: filter 0.3048 mm Al and 0.0254 mm Ti, 40 kV, 12 µA). For complete vessel analysis, the rotated stand was used. On the surface of each vessel, 24 lines were marked, and on each of them, 9 (vessel 1) or 8 (vessel 2) points were analyzed. For pottery-fragment analysis, the laboratory stand was used. The sample was placed on the spectrometer stand and oriented in correspondence with the original external and internal surfaces of the ceramic vessel and analyzed. When the sample was rotated, the analysis was repeated. The value of element concentrations was calculated as the mean of three repetitions. The quality of the XRF analysis procedure was checked using Bruker test samples for the Mudrock calibration.

For the analysis of the mineral composition of samples, the FTIR-Alpha spectrometer (Bruker AXS, USA) was used. The basic technical parameters of the analytical system were a spectral range of 400–4000 cm^−1^, spectral resolution of 4 cm^−1^; the system was equipped with an Attenuated Total Reflectance (ATR) module. The milled sample was measured three times. The obtained spectra were analyzed both manually and using the infrared spectra library from the RRUFF FTIR database [58]. Calibration, drift correction, and data quality control were provided automatically by the instrument software. The peak fitting was performed using OriginPro^®^ 2025b software (OriginLab Corporation, Northampton, MA, USA).

In iron forms determination by colorimetry, the photometer Slandi LF300 (Slandi, Żory, Poland) was used. The photometer allowed measurements at the selected wavelengths (470 and 520 nm) with the blank correction. As the reference hyphenated system, HPLC-ICP hrOES was used. The HPLC system was constructed from an HPLC pump, the ProStar 215 HPLC Solvent Delivery Module (Varian, Macquarie Park, Australia), and a cation–exchange HPLC column, Dionex IonPac CS5A, 250 mm × 4.0 mm i.d., resin particle size 5 µm (Thermo Fisher Scientific, Waltham, MA, USA). The system was constructed using PEEK (polyetheretherketone) tubing and a 200 µL sample loop. The PDCA mobile phase flow rate was 2 mL min^−1^. As a chromatographic detector, the inductively coupled plasma high-resolution optical emission spectrometer, ICP hrOES PlasmaQuant 9100 Elite (Analityk Jena, Jena, Germany), was used. The following conditions were used: analytical wavelength 259.940 nm, axial plasma view, radio-frequency (RF) power 1.20 kW, plasma gas flow 12.0 L min^−1^, nebulizer gas flow 0.50 L min^−1^, and auxiliary gas flow 0.5 L min^−1^. Due to the lack of certified standard materials for iron forms, the standard addition method was used for measurement quality control. The recovery in the range 80–120% was acceptable. The accuracy of the results obtained by the reference method was in the same range.

The analysis of the experimental data was performed by using Statistica 13.1 software (StatSoft, Tulsa, OK, USA). In the descriptive statistics, the basic parameters were defined: minimal and maximal values, median, mean, standard deviation, and relative standard deviation. The next step of the data analysis was exploration analysis. Multidimensional statistical analysis (principal components analysis, PCA) was performed to identify the individual differences in the chemical composition of the pottery samples. For all statistical tests, the probability value of *p* = 0.05 was applied.

### 3.4. Sample Preparation and Analytical Procedures

The undamaged vessels for XRF measurement were cleaned of dust using plastic tools; next, the vessel surfaces were cleaned with a soft brush and washed with methanol.

The large fragment of pottery was divided into 30 samples (about 1–2 cm in diameter—Figure S3) by plastic tools. After XRF analysis, samples were ground in an electric agate mortar and sieved through a 0.02 mm (No. 10 mesh) plastic sieve. The extraction by hydrochloric acid was prepared according to the following methodology. An accurately weighed 1.00 ± 0.01 g of sample was put into a flask with a reflux condenser and 20 mL of hydrochloric acid solution (2 mol L^−1^) and was heated to approximately 80 °C for 30 min. After cooling, the solution was filtered with the use of a paper filter (washed previously using 200 mL of ultra-pure water) and finally diluted with water to a volume of 50.0 mL. Hydrochloric acid extracts were analyzed using UV-Vis and HPLC-ICP hrOES (for selected samples as a reference method) to determine the concentration of iron forms. The content of Fe(II) and Fe(III) forms was determined using colorimetry. Fe(III) was determined at a wavelength of 470 nm after reaction with thiocyanate (in the hydrochloric acid (pH < 2.0)), and Fe(II) was determined at a wavelength of 520 nm after reaction with 2,2′-bipirydyl (in the acetate buffer (pH 4.5)). For selected samples, the hyphenated technique HPLC-ICP hrOES was used as the reference method.

## 4. Conclusions

Studies indicate similar variation in elemental content across entire ceramic vessels, vessel fragments, and sets of vessel fragments. Variability in elemental content is an integral and “natural” characteristic of ceramics. Variability in elemental content within a single archaeological artifact must therefore be considered when evaluating the variability of the chemical composition of a collection of multiple objects. A lack of knowledge about the heterogeneity of the material being examined can lead to overinterpretation of results and the misidentification of objects potentially differing in chemical composition from the studied population. In archaeological ceramics research, this can lead, for example, to misclassifying local pottery as imported. However, it should be emphasized that a similar risk applies to the analysis of any materials characterized by heterogeneity in the examined feature. The research presented has highlighted the problem of interpreting the results of archaeological ceramic analyses. While focusing on the accuracy of analytical results, the scatter of analytical values resulting from the natural properties of the material (e.g., its inhomogeneity) is often overlooked. This research indicates that it is necessary to consider the inhomogeneity of ceramic composition when interpreting analytical results—drawing conclusions about imports or workshop differences must be approached with extreme caution. Most importantly, studies of small (several dozen fragments) collections of ceramic objects should be treated as “case studies” and not as a basis for general considerations.

## Figures and Tables

**Table 1:** Selected results of XRF measurements are provided for two vessels. (vessel 1 *n* = 237, vessel 2 *n* = 155), mg/kg.

**Vessel 1**	**Si**	**Al**	**K**	**Fe**	**Ca**	**Ti**	**Ba**	**Mn**
min	73,600	7410	42,600	24,700	17,700	4830	715	332
max	345,000	114,000	68,500	81,100	37,600	10,300	16,500	847
median	305,000	92,100	56,400	48,600	25,000	7750	7790	523
mean	290,000	89,100	56,200	47,300	25,300	7680	7820	537
SD	55	18,700	5340	8370	3470	951	2650	107
RSD%	19	21	10	18	14	12	34	20
max/min	4.7	15	1.6	3.3	2.1	2.1	23	2.5
**Vessel 2**	**Si**	**Al**	**K**	**Fe**	**Ca**	**Ti**	**Ba**	**Mn**
min	25,600	7810	2640	15,200	3780	1550	100	185
max	226,000	71,900	25,000	57,100	28,700	7620	4340	468
median	142,000	36,500	14,800	45,300	18,600	5420	1400	302
mean	139,000	36,700	14,700	44,500	18,400	5360	1580	305
SD	45,500	13,200	4850	6170	3960	916	1070	52
RSD%	33	36	33	14	22	17	68	17
max/min	8.8	9.2	9.5	3.8	7.6	4.9	43	2.5

**Table 2:** Selected results of XRF measurements are provided for a sample of one fragment of pottery divided into 30 fragments: outer (*n* = 30) and inner (*n* = 30) parts, mg/kg.

**Outer Part**	**Si**	**Al**	**K**	**Fe**	**Ca**	**Ti**	**Ba**	**Mn**
min	127,000	35,000	6500	26,900	2110	1620	11	316
max	240,000	84,200	22,400	53,200	8810	4030	427	1290
median	204,000	56,400	17,900	46,300	5960	3190	103	512
mean	201,000	57,300	16,800	44,900	5780	3070	119	588
SD	26,400	9580	3710	6440	1610	545	71	229
RSD%	13	17	22	14	28	18	60	39
max/min	2.0	2.4	3.4	2.0	4.2	2.4	39	4.1
**Inner Part**	**Si**	**Al**	**K**	**Fe**	**Ca**	**Ti**	**Ba**	**Mn**
min	170,000	51,800	9732	23,400	1950	2200	48	175
max	278,100	84,600	15,100	40,800	5030	4330	1350	969
median	211,000	65,800	12,851	33,300	4230	3480	180	272
mean	215,000	66,800	12,788	33,900	4000	3350	237	330
SD	28,000	7490	1396	4280	805	552	160	168
RSD%	13	11	11	13	20	16	67	51
max/min	1.6	1.6	1.5	1.7	2.6	2.0	28	5.5

**Table 3:** Selected results of XRF measurements for the samples of Pre-Roman Iron Age pottery (*n* = 100) based on [2], mg/kg.

	Si	Al	K	Fe	Ca	Ti
min	234,000	99,000	10,900	20,600	4510	2890
max	308,000	177,000	30,100	73,400	44,600	5200
median	266,000	144,000	19,100	46,500	14,900	3730
mean	268,000	143,000	18,700	46,600	16,900	3810
SD	15,200	13,200	3700	11,500	8090	451
RSD%	6	9	20	25	48	12
max/min	1.3	1.8	2.8	3.6	9.9	1.8

## Data Availability

The original contributions presented in this study are included in the article/Supplementary Material. Further inquiries can be directed to the corresponding author.

## Supplementary Materials

The following supporting information can be downloaded at: https://www.mdpi.com/article/10.3390/molecules30244732/s1, Figure S1. Exemplary map (for 1/24 of the vessel surface) of concentration (XRF measurements) of selected elements in first vessel. Figure S2. Exemplary map (for 1/24 of the vessel surface) of concentration (XRF measurements) of selected elements in second vessel. Figure S3. Fragment of vessel divided into 30 samples. Figure S4. Microscopy view of pottery fragment.

## Abbreviations

ATR	attenuated total reflectance
FTIR	fourier transform infrared spectroscopy
HPLC-ICP hrOES	high-performance liquid chromatography–inductively coupled plasma high-resolution optical emission spectrometry
PCA	principal components analysis
PDCA	pyridine–2,6–dicarboxylic acid
PEEK	polyetheretherketone
UV-Vis	ultraviolet-visible spectrophotometry
XRF	X-ray fluorescence spectrometry
