# Supplementary material for: Multi-Techniques Analysis of Archaeological Pottery—Potential Pitfalls in Interpreting the Results
Source: Molecules. 2025 Dec 10;30(24):4732. doi: 10.3390/molecules30244732 (PMC12736328; doi:10.3390/molecules30244732)

Multi-techniques analysis of archaeological pottery - potential pitfalls in interpreting the results

Figure S1. Exemplary map (for 1/24 of the vessel surface) of concentration (XRF measurements) of selected elements in first vessel

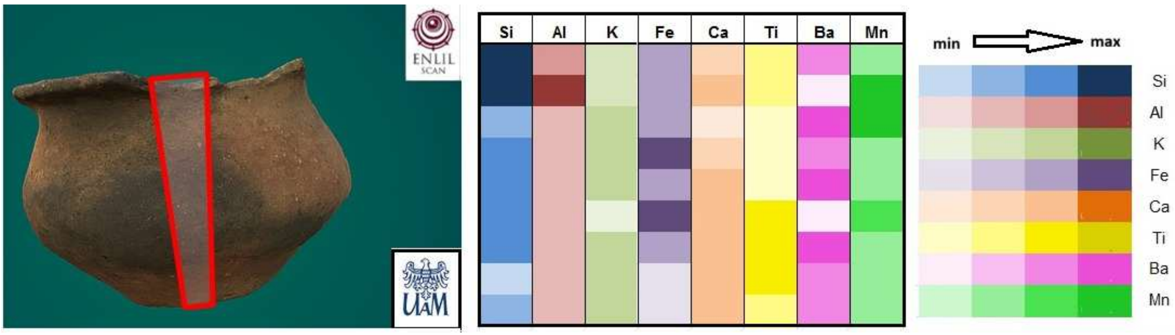

Figure S2. Exemplary map (for 1/24 of the vessel surface) of concentration (XRF measurements) of selected elements in second vessel

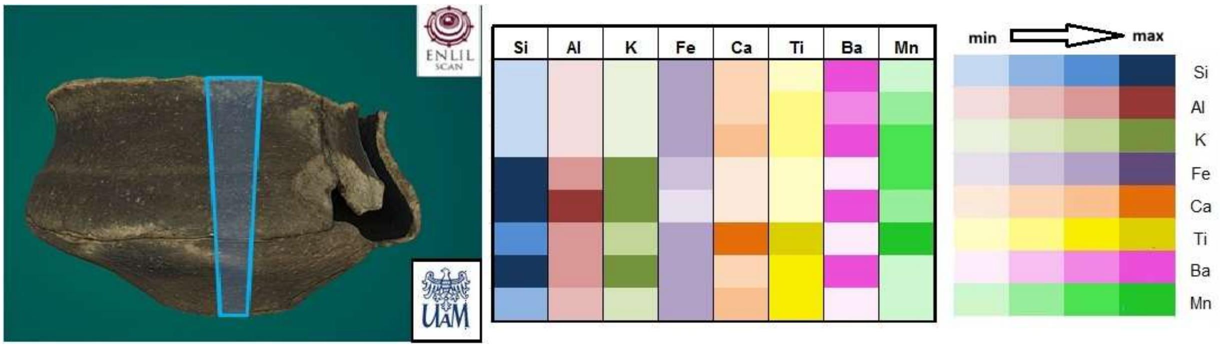

Figure S3. Fragment of vessel divided into 30 samples

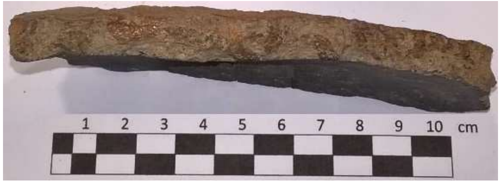

outer side inner side

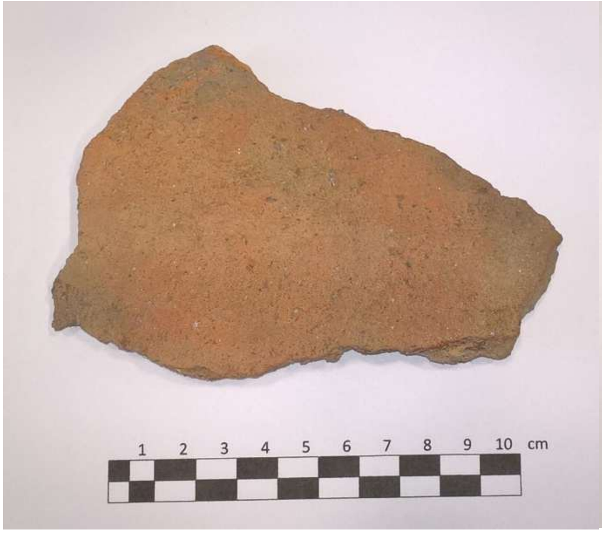

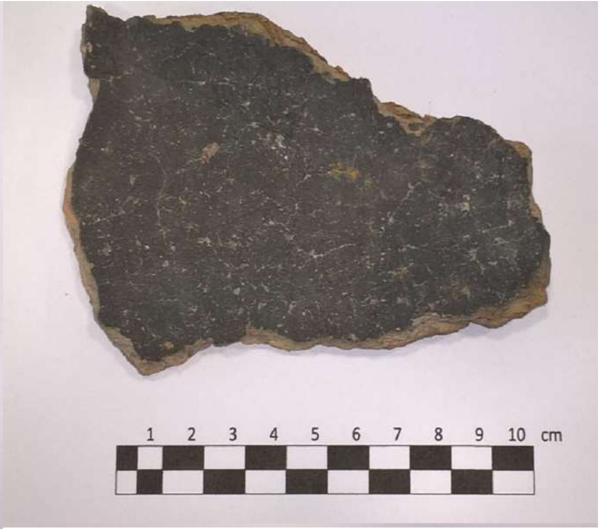

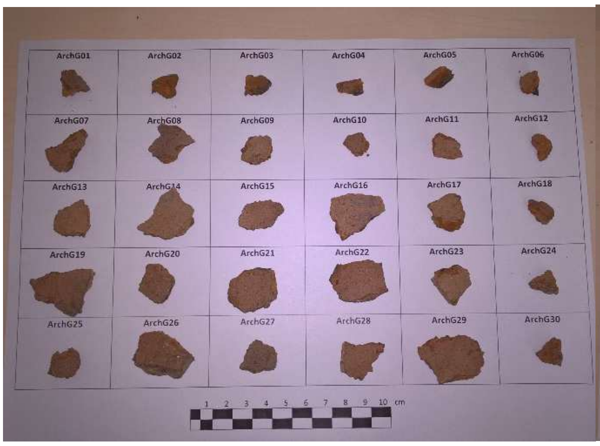

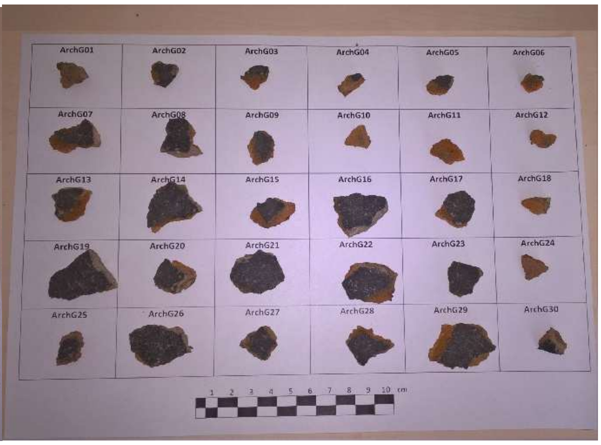

Figure S4. Microscopy view of pottery fragment

(zoom 5x)

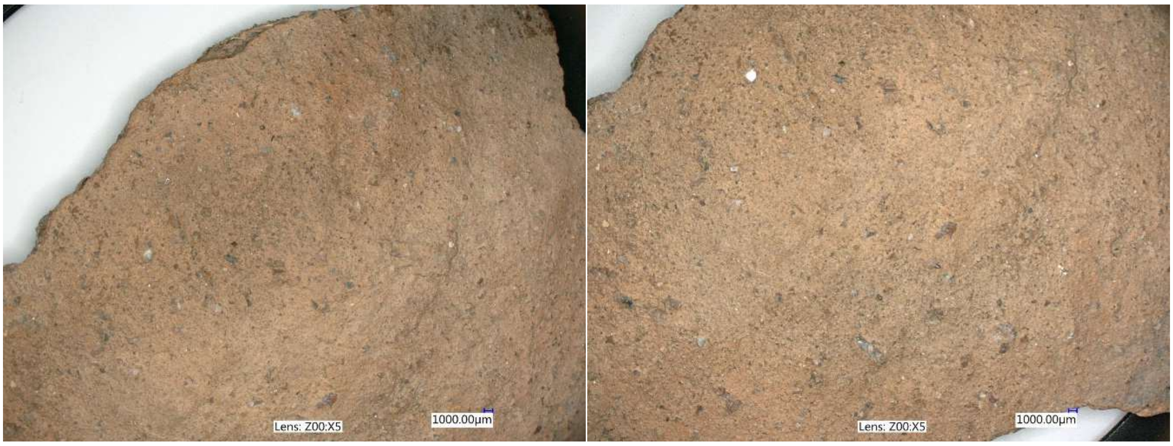

(zoom 50x)

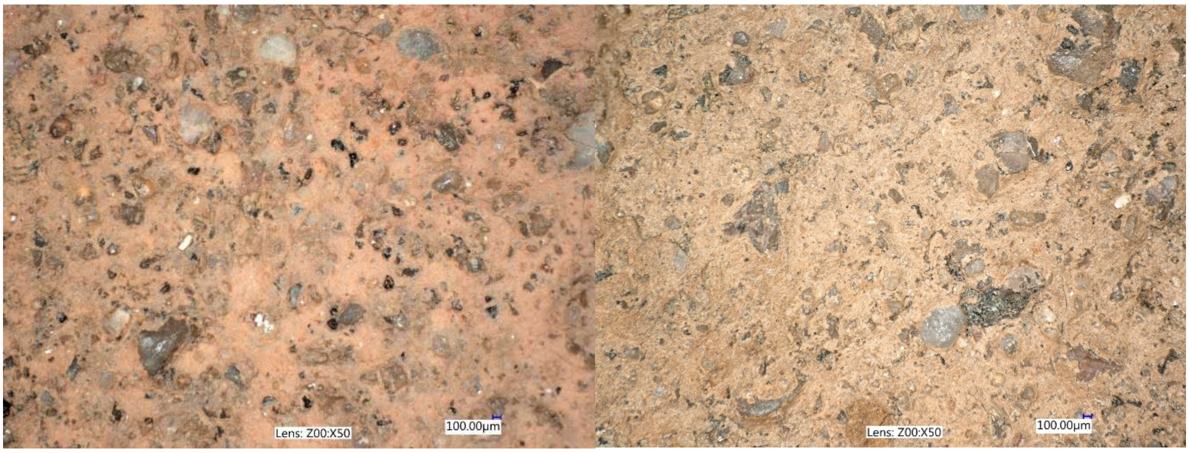

Supplement: Supplementary file 1 [file molecules-30-04732-s001.zip › molecules-4018723-supplementary.pdf]
